# TGCT of the shoulder – a case series and literature review

**DOI:** 10.1007/s00402-026-06237-z

**Published:** 2026-03-09

**Authors:** Josefa Stadelmeier, Filip Bijeljac, Alexander Klein, Felix Winden, Paul Reidler, Hans Roland Dürr

**Affiliations:** 1 Orthopaedic Oncology, Department of Orthopaedics and Trauma Surgery, University Hospital, LMU Munich, Campus Großhadern, Munich, Germany; 2 Department of Radiology, University Hospital LMU Munich, Campus Großhadern, Munich, Germany

**Keywords:** Tenosynovial giant cell tumor, Pigmented villonodular synovitis, Shoulder, Open resection, Arthroscopy, Recurrence

## Abstract

**Introduction:**

Tenosynovial giant cell tumor (TGCT), also known as pigmented villonodular synovitis (PVNS), involving the shoulder is extremely rare and can present with a challenging clinical course. Due to the complex anatomy of the shoulder, both diagnosis and treatment are demanding. This study aims to evaluate the diagnostic and therapeutic management of shoulder TGCT based on a case series and a review of the literature.

**Materials and methods:**

Between 2005 and 2021, four patients (2 females, 2 males) with shoulder TGCT (1 localized, 3 diffuse) underwent surgical treatment at our institution. The minimum follow-up was 39 months (range: 39–233 months). Functional outcomes were assessed using the Disabilities of the Arm, Shoulder and Hand Questionnaire (DASH), the Oxford Shoulder Score (OSS), and the Short Form 36 Health Survey (SF-36). Additionally, a literature review of 65 studies comprising 108 patients was performed.

**Results:**

Treatments consisted of arthroscopic and open resections, tendon repair, adjuvant radiosynoviorthesis, and radiotherapy, as appropriate. All patients were recurrence-free at the last follow-up, except for one with stable residual disease after radiotherapy. The mean interval from symptom onset to diagnosis was 23.6 ± 28.1 months. In the literature cohort, the mean patient age was 50.3 ± 19.7 years, with a nearly equal gender distribution. Diffuse TGCT was more common (66.1%) than nodular TGCT (33.8%). Treatment was primarily surgery, arthroscopic (53.2%) or open (42.9%), with adjuvant therapies applied in 9.3% of cases. After a mean follow-up of 37.6 ± 36.2 months in 67 cases, diffuse in 44.8%, nodular in 19.4% (no data regarding TGCT-type in 35.8%) the reported recurrence rate was 10.6% and 4.5% remained with residual disease.

**Conclusions:**

TGCT of the shoulder remains a rare and complex condition requiring individualized treatment strategies. Arthroscopic and open resections are the mainstays of therapy, while the role of adjuvant treatments should be carefully considered. Given the risk of recurrence, follow-up is essential. Further studies are needed to establish standardized treatment protocols and evaluate long-term outcomes.

**Level of evidence:**

Level IV (Case series with no comparison group).

## Introduction

### Background

**Tenosynovial giant cell tumor** (TGCT), formerly known as Pigmented Villonodular Synovitis (PVNS), is a rare, benign proliferative disorder of the synovium, the tendon sheath and the bursa [1]. Typically, TGCT presents as a monoarticular process and predominantly affects the knee joint, hip, ankle, shoulder, elbow as hands and feet. Two types of TGCT are recognized: the nodular-type TGCT (N-TGCT) and the diffuse-type TGCT (D-TGCT) [2].

### Epidemiology

Epidemiological data on TGCT are limited and varied, making comparisons challenging. In nationwide studies, the incidence rates of N-TGCT ranged around 11 to 34 per million. For D-TGCT, incidence rates were reported between 5 and 8.4 per million [3–5]. 

There is inconsistency in the data concerning the gender distribution of pigmented villonodular synovitis. While some studies indicate that males and females are affected at similar rates, others suggest a slight predisposition in females, specifically in cases of N-TGCT [3, 6]. TGCT predominantly presents between 35 and 50 years of age [5, 6].

### Pathogenesis

The pathogenesis of TGCT remains unclear, with theories ranging from chronic inflammatory processes to neoplastic etiology [7, 8]. Last is at present the commonly accepted pathogenesis, hence the name was changed from PVNS to TGCT [9, 10].

The most frequently suggested associations include chronic recurring trauma, intra-articular hemorrhage, and persistent inflammation [11, 12]. West et al. hypothesized that a chromosomal translocation involving the 1p13 locus may contribute to the underlying pathogenesis [13]. Colony-stimulating factor-1 (CSF-1) overexpression promotes the formation of aberrant cellular aggregates, contributing to synovial soft tissue hyperplasia and thus defining the clinical and radiological disease [14].

### Imaging and clinical presentation

TGCT is one of the few soft-tissue conditions with a nearly pathognomonic magnetic resonance imaging (MRI) appearance, characterized by synovial hypertrophy, low signal intensity and a “blooming artifact” [15–17]. This artifact, caused by iron in hemosiderin deposits, appears as notable signal loss on both T1- and T2-weighted MRI [18]. While N-TGCT presents most commonly with a painless swollen joint and MRI shows a well-circumscribed soft tissue mass [19], D-TGCT presents with a painful swollen joint, often with reduced mobility. On MRI an ill-defined soft-tissue mass is visualized [15]. In some cases impingement is the leading symptom [20].

### Clinical, histological and molecular profile

Clinically, N-TGCT typically presents as a well-circumscribed, nodular mass causing painless swelling or mechanical symptoms (e.g., locking, impingement), with slow growth and low risk of local invasion. In contrast, D-TGCT manifests as an infiltrative synovial process with painful swelling, effusions, stiffness, and progressive joint destruction over time, often requiring repeated interventions if untreated. N-TGCT rarely progresses to diffuse form, while D-TGCT may extend intra- and extra-articularly [5]. 

N-TGCT typically shows mononuclear histiocyte-like cells, multinucleate giant cells, lymphocytes, and hemosiderin deposits, with moderate mitotic activity [15, 21]. D-TGCT displays a more infiltrative pattern with higher mitotic rates, a variable presence of giant cells, and occasional sarcoma-like features [22].

Molecularly, both forms are clonal neoplasms driven by CSF1 overexpression, often due to genomic rearrangements such as COL6A3::CSF1. This overexpression promotes tumor growth via paracrine recruitment of inflammatory cells and forms the basis for targeted therapy with CSF1R inhibitors [23–26]. 

### Treatment strategies

In both N-TGCT and D-TGCT, the decision to pursue active treatment should carefully weigh the potential for cure against the typically benign nature of TGCT, its sometimes slow progression, the risk of local recurrence (LR) after complete resection, and the possibility of surgery-related complications [5]. Active surveillance should be considered as approach for asymptomatic patients [5].

The standard of care for symptomatic TGCT is surgical intervention, provided it can be performed with acceptable morbidity [5]. For N-TGCT, marginal excision is sufficient, whereas D-TGCT requires extensive synovectomy to achieve local disease control and optimize functional outcomes. Surgical access may be achieved via an open anterior approach or arthroscopically, depending on tumor extent and anatomical considerations [27]. In cases of extensive rotator cuff pathology or advanced joint destruction, rotator cuff repair or shoulder arthroplasty in combination with complete synovectomy may provide effective symptom relief and functional restoration.

### Adjuvant therapies

Current evidence is insufficient to support radiotherapy as a standard treatment for TGCT in neoadjuvant, adjuvant, or recurrent settings. Radiation therapy can be in very selected cases an effective supplementary treatment, particularly where complete synovectomy is not feasible. It helps to prevent recurrences and preserves joint function with minimal toxicity [28, 29]. Other adjuvant therapies may include radiosynoviorthesis (RSO) and cryotherapy [30]. Both are at the moment considered as not beneficial.

### Systemic treatment

In current trials, CSF1R pathway inhibitors such as Pexidartinib, Imatinib, Nilotinib, Vimseltinib, and monoclonal antibodies such as Emactuzumab have demonstrated effectiveness in TGCT, leading to significant tumor reduction, symptom relief, improved function, and sustained disease control [31–35]. Systemic treatment with CSF1R inhibitors may be considered when symptomatic TGCT cannot be surgically removed with acceptable morbidity and symptoms remain difficult to manage, particularly if disease progression is likely to impair quality of life (QoL) [5].

### Study

In 1999, only 25 cases had been described in the English literature, managed by open synovectomy in all cases [36]. In 2017, Noailles et al. provided a literature review including 6 studies with 46 patients [36–42]. In this study, we present a series of four patients with TGCT of the shoulder, illustrating the heterogeneous clinical presentations, diagnostic challenges, and therapeutic outcomes associated with this rare entity. Additionally, we conducted a comprehensive review of the existing literature on shoulder TGCT, including 65 studies comprising 108 patients. Through the analysis of our cases and the available evidence, we aim to expand the limited knowledge on shoulder TGCT by providing insights into its clinical and radiological features, while underscoring the importance of timely diagnosis and appropriate management to optimize patient outcomes.

## Materials and methods

### Patient selection

Between March 2005 and May 2021, 4 patients (2 female, 2 male; mean age 38.8 ± 21.4 years) were diagnosed with TGCT of the shoulder—three with diffuse-type and one with localized-type (localized-type will be called nodular-type throughout the text). The right (dominant) shoulder was affected in three cases, the left in one. All patients underwent preoperative MRI and intraoperative histopathological confirmation by our Department of Pathology. Symptoms had been present for an average of 32.5 months (range: 12–60 months) before diagnosis. Surgical treatment was performed in all cases, including one rotator cuff repair. Two patients received adjuvant RSO. The mean follow-up period was 90 ± 95 months, with a minimum of 39 months. Patient characteristics are summarized in Table 1.

**Table 1:** Patient characteristics

Patient No.		Age/ Sex	Duration of preoperative Symptoms (mo)	TGCT type	Torn Rotator Cuff Tendon	Adjuvant therapy	Recur-rence	Follow-Up (mo)
**1**	54/f		31	nodular	SSP	no	no	40
**2**	24/f		27	diffuse	-	no	no	39
**3**	17/m		12	diffuse	-	RSO	yes	48
**4**	60/m		60	diffuse	-	RT	stable disease	233

(abbreviations: f = female; m = male; mo = months; TGCT = Tenosynovial Giant Cell Tumor; SSP = Supraspinatus Tendon; RSO = radiosynoviorthesis; RT = Radiotherapy)

### Surgical technique

Patients 1, 2, and 4 were positioned in a 30° beach-chair setup, while patient 3 was placed supine with the upper body elevated 30°; all underwent general anesthesia. The arm was positioned freely in all cases. Patient 1 initially underwent diagnostic shoulder arthroscopy with supraspinatus tendon repair and bony decompression, followed by open N-TGCT resection via the deltopectoral approach. Patients 2, 3, and 4 underwent primary open resections of D-TGCT using the deltopectoral approach. Patient 4 required a planned two-stage procedure; the second stage involved repositioning in left lateral decubitus and dual access via a medial scapular and subspinal incision.

### Pathology

Tissue samples from synovial tissue were stained with hematoxylin and eosin, Elastica-van-Gieson (EvG) and iron stain and reviewed under a light microscope by a pathologist who specialized in musculoskeletal pathology.

### Postoperative rehabilitation

Drains were removed within 1–3 days postoperatively. Patients 1–3 received interscalene plexus blocks for 4–5 days, after which all patients maintained adequate pain control according to WHO classification levels 1–2. Shoulders were immobilized in an arm sling for 4–6 weeks, with passive glenohumeral mobilization up to 60° in abduction and flexion. From week 4, assisted active movements up to 90° were introduced, excluding external rotation. Beginning in week 7, exercises were progressively intensified beyond 90°, with rotation as tolerated. The goal was full shoulder mobility by 12 weeks, with return to sports permitted no earlier than week 12.

In the 1st year, MRI follow-up checks were carried out every 3 months, every 6 months in the 2nd year, then annually for 5 years.

### Outcome instruments

The patient related outcome measures (PROMs) of this study included the Disabilities of Arm, Shoulder and Hand Questionnaire (DASH), the Oxford-Shoulder-Score (OSS) and the Short Form 36 Health Survey (SF-36). The total DASH score ranges from 0 to 100, where a score of 0 indicates an excellent functional outcome. The OSS ranges from 0 to 48, with a score of 48 representing the optimal functional outcome. The SF-36 health survey scores range from 0 to 100, where a score of 100 reflects excellent health and the highest quality of life. These PROMs were collected postoperatively at the time of the last follow-up. The rest of the data were retrieved from the patients’ medical records.

### Literature search strategy and selection criteria

A comprehensive literature search was conducted to identify all published studies reporting on TGCT of the shoulder. The search was performed using PubMed, google scholar, Web of Science from their inception to 15.02.2025. The following search terms were used in combination with Boolean operators: “tenosynovial giant cell tumor” OR “TGCT” OR “pigmented villonodular synovitis” OR “PVNS” OR ″giant cell tumor of the tendon sheath″ AND “shoulder”.

Inclusion criteria were: (1) studies reporting on TGCT of the shoulder; (2) clinical studies, case series, or case reports; (3) abstracts published in English. Exclusion criteria included: (1) studies focusing on TGCT of other joints without specific mention of the shoulder; (2) review articles without original patient data.

Titles and abstracts were screened by 2 independent reviewers, followed by a full-text assessment for eligibility. Any disagreements were resolved by consensus or consultation with a third reviewer. References of included articles were manually screened to identify additional relevant studies.

### Statistical analytic tools

Analysis was performed with Microsoft Excel 2016. No statistical hypothesis tests were performed due to the small sample size (*n* = 4). Results are presented descriptively using means ± standard deviations, ranges, and confidence intervals where appropriate to characterize the patient cohort and outcomes.

## Results

### Case 1

A 54-year-old female presented with a 2-year and 7-month history of progressive, movement-dependent left shoulder pain and restricted mobility without prior trauma. The pain was exacerbated by activity and present at rest and during the night. Conservative treatment, including physiotherapy and a corticosteroid injection, was ineffective.

**Fig. 1:**
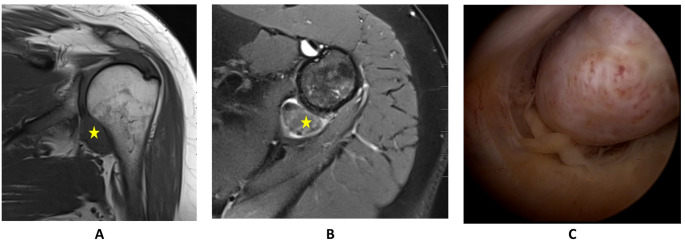
MRI scans and arthroscopic images of TGCT of the shoulder in patient 1. **A**: T1w coronal view of the left shoulder, depicting a mass in the lower recess. **B**: T2w axial view of the left shoulder, depicting a mass in the dorsal lower recess. **C**: Arthroscopic view of a mass in the dorsal lower recess of the left shoulder

MRI revealed a solid intra-articular mass with hemosiderin deposits in the shoulder recess, suggestive of TGCT (Fig. 1), along with AC joint osteoarthritis, a supraspinatus tendon tear with footprint cyst formation, and early degenerative changes of the glenohumeral joint. Clinically, TGCT appeared asymptomatic, with the symptoms attributed primarily to the rotator cuff pathology. Examination showed tenderness over the AC joint, subacromial space, and bicipital groove. Active range of motion included 160° flexion, 100° abduction, internal rotation to L2, and 30° external rotation. Impingement signs and supraspinatus and AC joint tests were positive.

Arthroscopy revealed articular-sided partial rupture of the SSP (Ellman III), SLAP I lesion, subacromial bursitis, acromioclavicular joint arthrosis, bony subacromial impingement, and a cherry-sized nodular lesion in the lower recess (Fig. 1). Arthroscopic synovectomy, SLAP debridement, subacromial bursectomy, lateral clavicle resection, supraspinatus tendon repair using a SpeedScrew anchor (Smith & Nephew GmbH, Hamburg, Germany) and bony subacromial decompression were performed. In open surgery, the pedunculated, dorsally luxated N-TGCT was removed in total. Histopathology confirmed TGCT. Three years and four months postoperatively, the patient remained free of recurrence with excellent functional outcome and full range of motion (PROMs in Tables 2 and 3).

### Case 2

**Fig. 2:**
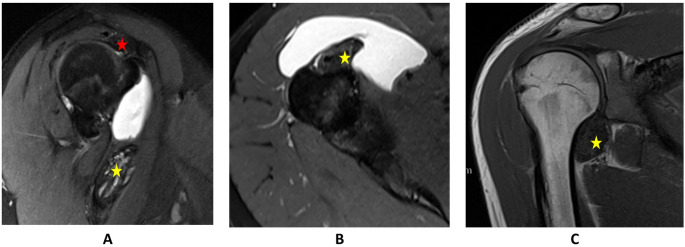
MRI scans of TGCT of the shoulder in patient 2. **A**: sagittal PD tse fs TE 47 of the right shoulder; **B**: axial PD tse fs TE 47 of the right shoulder; **C**: coronal T1 TSE of the right shoulder; The star marks the masses of TGCT around the bicpes tendon (yellow) and subacromial (red)

A 24-year-old female patient reported right shoulder pain for two years and three months, treated conservatively. The pain was intermittent and worsened with specific movements. For two months, she noticed a lump in the upper arm accompanied by increased pain, which later subsided. Further diagnostic workup, including MRI was initiated. A diagnosis of diffuse TGCT involving the long biceps tendon and anterior shoulder joint was confirmed (Fig. 2).

She underwent marginal resection of the TGCT lesion. The patient was positioned in a beach chair position with a deltopectoral surgical approach. Intraoperative findings included extensive TGCT masses along the long head of the biceps tendon, requiring transection and refixation of the subscapularis tendon. A solid TGCT lesion caudally was also noted, pedunculated and attached to the capsule. Histopathology confirmed TGCT.

Three years and three months postoperatively, the patient remained free of recurrence with excellent functional outcome (PROMs in Tables 2 and 3).

### Case 3

**Fig. 3:**
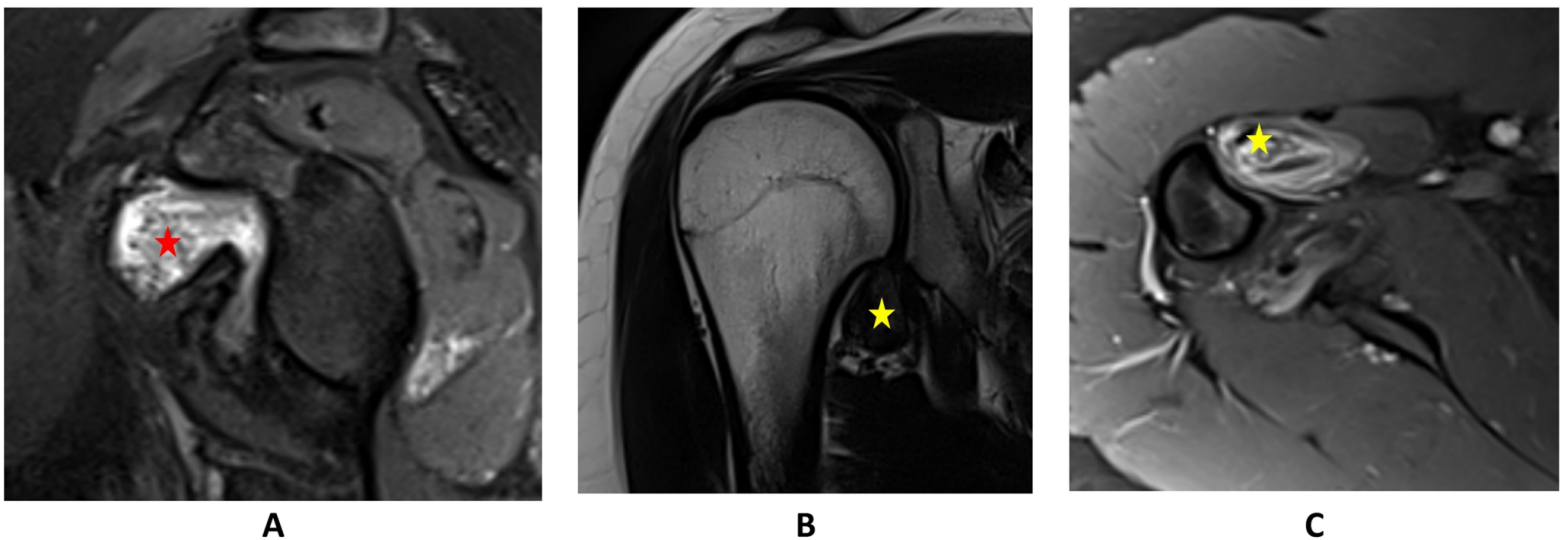
MRI scans of TGCT of the shoulder in patient 3. **A**: PDFS sagittal of the right shoulder: red star marks the mass below the coracoid. **B**: T2 coronal of the right shoulder: yellow star marks the mass in the lower recess. **C**: T1FS axial view of the right shoulder: yellow star marks the mass in the lower recess

An 18-year-old male presented with progressive, atraumatic right shoulder pain over one year, localized intra-articularly and radiating to the proximal humerus. The discomfort was described as intra-articular, radiating to the proximal humerus, and unrelated to mechanical load. MRI revealed a suspected TGCT in the inferior recess and below the coracoid process (Fig. 3). Open synovectomy via a deltopectoral approach confirmed TGCT histologically. Postoperative recovery was initially uneventful.

Seven months postoperatively, MRI revealed a LR, necessitating a repeat open synovectomy and subsequent RSO. Despite initially unremarkable follow-up imaging, progressive lesion growth was noted 1.5 years later. The patient was clinically symptom-free. A third surgical intervention—arthroscopic total synovectomy—was performed three years after the initial recurrence, revealing extensive brownish synovitis and mild cartilage damage (grades I–II). The arthroscope was also repositioned ventrally in order to achieve a dorsal synovectomy of the lower recess. Histopathology again confirmed TGCT.

Postoperative care included free mobilization and a second RSO. The patient remained symptom-free with no radiological evidence of recurrence at one-year follow-up. One year later, there was no evidence of recurrence (PROMs in Tables 2 and 3).

### Case 4

**Fig. 4:**
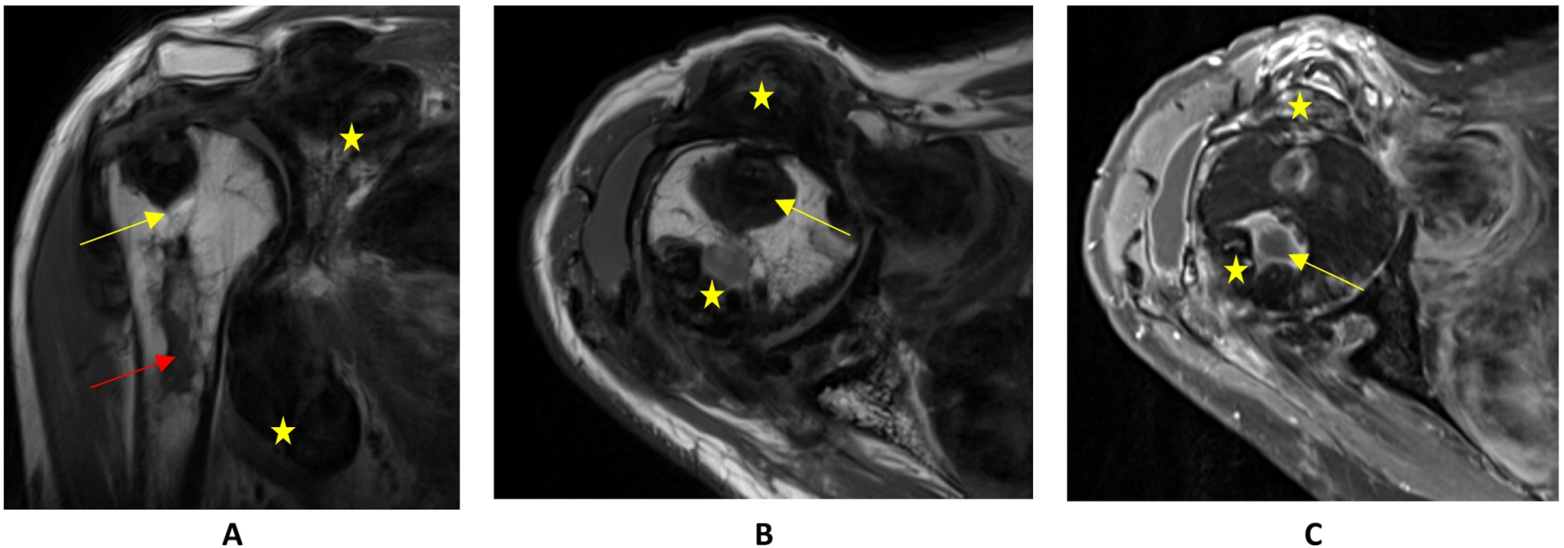
MRI scans of TGCT of the shoulder in patient 4. **A**: T1 TSE coronal: cystic components (marked by a yellow arrow). Destruction of the humeral head and erosions in the subcapital humeral shaft (marked by a red arrow). The lesions extend subacromial and to the medial thoracic wall (exemplified by yellow stars). **B**: T1 TSE axial: hypointense nodular structures (exemplified by yellow stars) with individual cystic components (marked by a yellow arrow). **C**: T1 TSE axial with contrast fluid: hypointense nodular structures (exemplified by yellow stars) with individual cystic components (marked by a yellow arrow) with increased absorption of contrast medium

A 61-year-old male patient experienced right shoulder pain for at least six years, progressively worsening over the past six months. Imaging, including X-rays and MRI, revealed extensive TGCT extending into the thoracic wall, involving the humeral shaft with intraosseous components, and encasing neurovascular structures without infiltration (Fig. 4).

The patient underwent open anterior synovectomy, tumor resection, and intraosseous curettage. This patient showed very pronounced TGCT findings: a large tumor extending subscapularly, a broad infiltration of the dorsal joint space extending far distally along the humerus. In addition, there was an extensive infiltration of the long head of the biceps tendon on the ventral side, destroyed to such an extent that it tore when pulled with a finger. Besides, the TGCT infiltrated the proximal medullary cavity in the bicipital sulcus. Here, the window was opened with the Luer and extensively curetted. Ultimately, the lesions could be completely removed ventrally, but there were still tumor parts in the area of the subscapularis muscle and the dorsal humerus, which were then removed in a planned second surgery six weeks later. Histopathology confirmed TGCT.

Postoperative care included immobilization in a Gilchrist brace for four weeks, and limited shoulder movement. Postoperative radiation therapy followed due to remaining tumor tissue.

MRI surveillance continued at regular intervals, revealed stable disease without significant progression over subsequent years. 19 years and 5 months after the initial diagnosis, the patient maintained functional mobility with mild restrictions (PROMs in Tables 2 and 3).

#### Outcome of DASH, OSS and SF-36

All scores for each patient and means are shown in Tables 2 and 3.

**Table 2:** Individual Oxford-Shoulder-Score (OSS) and Score of the Disabilities of Arm, Shoulder and Hand Questionnaire (DASH), including mean and standard deviation (SD) for the patient collective at a median follow-up of 44 months (range 39–233 months)

Patient no.	OSS	DASH	DASH Sport/ Instrument	Sport / Instrument	DASH Work	Work
**1**	47	0,83	0	Volleyball/ piano	0	Art teacher
**2**	48	0	0	Running, biking	0	Desk activity
**3**	47	0,83	25	Strength training	0	Computer scientist
**4**	41	21,67	12,5	Senior gymnastics	-	Pensioner
**Mean**	45,75	5,83	9,38		0,00	
**SD**	3,20	10,57	11,97		0,00	

**Table 3:** Individual scores of the Short Form 36 Health Survey (SF-36), including mean and standard deviation (SD) for the patient collective at a median follow-up of 44 months (range 39–233 months)

Patient no.	Physical Functioning	Role limitations due to physical health	Role limitations due to emotional problems	Energy/ fatigue	Emotional well-being	Social functioning	Pain	General health
**1**	80	0	0	30	40	88	0	35
**2**	100	100	100	80	96	100	100	100
**3**	95	100	100	80	88	100	90	40
**4**	55	100	100	50	88	75	78	60
**Mean**	82,50	75,00	75,00	60,00	78,00	90,75	67,00	58,75
**SD**	20,21	50,00	50,00	24,49	25,61	11,93	45,56	29,55

### Pathologic findings

Macroscopically, the excised tissues were variably orange-brown and gray-glassy.

Patient 1 (N-TGCT) showed a nodular lesion with oval nuclei, scattered disorganized giant cells, and hemosiderin-loaded macrophages, without atypia or necrosis.

Patients 2–4 (D-TGCT) demonstrated cell-rich synovial tissue with irregular giant cells, foam cells, hemosiderin deposits, and signs of old hemorrhage. Focal necrosis and marked bleeding were present, but no cellular atypia was observed.

#### Literature review

Shoulder TGCT is an exceptionally rare condition, with a total of 107 cases identified in the literature, including the four patients presented in this study. The sex distribution was nearly balanced, with a slight predominance of female patients (46.7%) over male patients (36.4%). The mean age at diagnosis was 50.3 ± 19.7 years, ranging from 5 to 84 years. The average duration of symptoms prior to diagnosis was 23.6 ± 28.1 months. TGCT subtype was specified in only 65 cases, of which 33.8% were classified as N-TGCT and 66.1% as D-TGCT. The affected side was reported in 58 cases and the left shoulder was affected in 34.5% of cases, the right shoulder in 58.6%, and bilateral involvement was reported in 6.9%. A concomitant rotator cuff tear was documented in 32.4% of patients, while bone involvement was reported in 30.6% of cases.

Surgical details were available for 77 cases: 42.9% underwent open surgery, 53.2% arthroscopic surgery, and 3.9% a combined approach. Rotator cuff repair was performed in 20.8% of cases, and rotator cuff debridement in 2.7%. Shoulder arthroplasty was performed in 12 cases (15.6% of surgical cases), including four hemiarthroplasties (HA), six reverse total shoulder arthroplasties (rTSA) (one of the rTSAs in a case of recurrence), one anatomic total shoulder arthroplasty (aTSA), and two cases where the type of prosthesis was not specified.

Adjuvant therapy was administered in 9.3% of cases (*n* = 10), including RSO in one patient (0.9%), radiotherapy in eight patients (7.5%), and osmium acid therapy in one patient (0.9%). The mean follow-up duration was 37.6 ± 36.2 months (range 6–233 months) in 67 cases. In those 44.8% were D-TGCT, 19.4% were N-TGCT and in 35.8% type of TGCT was not mentioned. In those 67 cases LR was reported in 10.6% (*n* = 7) and residual disease in 4.5% (*n* = 3). All reported recurrences and residual diseases were classifed as D-TGCT. Details of the literature review are depicted in Table 4.

**Table 4:** Results of the literature review of TGCT of the shoulder

source	number of patients	sex	age	duration of symptoms in months	nodular /diffuse	affected side	rotator cuff tear	bony involvement	type of surgery	cuff-repair	type of AP	adjuvant therapy	follow-up time (months)	recurrence	treatment of recurrence	residual lesion
Agrawal et al. [59]	1	m	38	4	d	r		yes	open				8	yes	RSO	yes
Broski et al. [60]	1	f	46			l										
Byers et al. [55]	1				d				open							
Cheng et al. [52]	1	m	20	36	n				open							
Chiang et al. [41]]	5	f					yes		AS				22,4	no		
		f					yes		AS				22,4	no		
		m					yes		AS				22,4	no		
		m					yes		AS				22,4	no		
		m					yes		AS				22,4	no		
Cho et al. [61]	1	m	21	3		r			AS + open				18	no		
Colak et al. [62]	1	f	49													
Costallat et al. [63]	1	f	15	12		r			AS				9	no		
Cotten et al. [64]	1	m	52	8	d				open							
Dias et al. [65]	1	f	59	1	n	l			open				24	no		
Dkhissi et al. [66]	1	m	68	36	d		yes		open				6	yes		
Dorwart et al. [43]	2	f	65	48	d		yes	yes		yes			114	yes	rTSA	
		m	56	9	d			yes	open		rTSA					
Du et al. [67]	1	f		12	d	l			open							
Elumogo et al. [68]	1	m	65	chronic		l										
Flandry and Norwood [69]	1	m	28		d				open				24	no		
Ganel et al. [70]	2	f	74	9	d		yes	yes	open				36	no		
		m	79	8	d		yes	yes	open				24	no		
Giannatos et al. [44]	1	f	50	24		l			AS				12	no		
Golge et al. [54]	1	m	75	chronic			yes	yes	open		rTSA					
Graf et al. [71]	1	f	80		d	b	yes		AS	yes						
Gumina et al. [40]	9	f	65,8	12		r	yes		AS	yes			27			
		f	65,8	12		r	yes		AS	yes			27			
		f	65,8	12		r	yes		AS	yes			27			
		f	65,8	12		r	yes		AS	yes			27			
		f	65,8	12		r	yes		AS	yes			27			
		f	65,8	12		r	yes		AS	yes			27			
		m	65,8	12		r	yes		AS	yes			27			
		m	65,8	12		l	yes		AS	yes			27			
		m	65,8	12		l	yes		AS	yes			27			
Ji et al. [51]	2	f	66	36	d	r	yes	yes	AS	yes		RT	24	no		
		f	71	120	d	r	yes	yes	AS	yes			36	no		
Johansson et al. [42]	4		35	72	d			yes			rTSA		24	no		
			35	72	d			yes			rTSA		24	no		
			35	24	n											
			35	12	d			yes					180	no		
Kho et al. [39]	1		64	4		b	yes		AS							
Konrath et al. [72]	1	m	41	36	d			yes	open				48	no		
Kwon et al. [73]	1	f	71	6	d	r		yes	open		HSA		7	no		
Lee, K. et al. [74]	1	f	27	0	n	l			AS				12	no		
Lee, S.-J. et al. [75]	1	m	64		d	b	yes	yes	AS							
Lee, M. et al. [76]	1	f	40		d	l		yes	open				43	no		
Levin and Gannon [77]	2	f	79	2	d				open							
		f	21	6	d			yes	open							
Lewis et al. [78]	1	m	58	0	n	r										
Li et al. [27]	6	f	45		n/d	r	yes		AS				92	yes	2 x AS (progress from N-TGCT to D-TGCT)	
		f	30		n	l			AS			RT	62	no		
		f	65		n	r	yes		AS				44	no		
		f	62		n	r	yes		AS				40	no		
		f	38		n	r			AS				28	no		
		m	64		n	r	yes		AS				46	no		
Liang et al. [79]	1					r										
Loh et al. [80]	1	m	46	6	n	r			AS + open				9	no		
Mahieu et al. [38]	2	f	30	96	n	l		yes	AS				96	no		
		f	53	36	d	r	yes	yes	AS			osmium acid	24	no		
Mankin et al. [81]]	4															
Mazabraud et al. [82]	4							yes								
								yes								
Mazabraud et al. [82]	1	f	17		d	r (mf)			conservative		RT		14	residual		yes
Mulier et al. [84]	1	m	64	12	d		yes	yes		yes			6	no		
Muller et al. [36]	1	m	16	4	n	l		yes	open							
Palmerini et al. [85]	2								open		AP					
									open		AP					
Pantazopoulos et al. [53]	1	m	36		d			yes	open							
Park et al. [49]	1		29		d		yes	yes	open		HSA		24	no		
Pereira et al. [20]	1	f	15	24	n	r			AS				12	no		
Petsatodis et al. [48]	2	f	60	12	d	r			open		HSA		48	no		
		m	63	6	d	l			open		HSA		60	no		
Popov et al. [86]	1	f	63	120	d	l		yes	open			RT	12	no		
Rajakulasingam et al. [87]	1	f	60		n											
Rezaee, A. et al. [88]	1	m	59		n											
Sawmiller et al. [89]	1	f	57	12			yes			yes			21	no		
Sayegh et al. [90]	1	f	34	20	d	l			AS							
Schwartz et al. [56]	2	m	55	36	n				open							
		m	58	90	d			yes	open		rTSA					
Seiler et al. [91]	1	m	55	60												
Selby et al. [92]	1	m	58		n	r			open				60	no		
Serra et al. [29]	1	m	74	8	d		yes	yes	AS			RT				
Sher et al. [93]	1	f	18		n			yes								
Simmer et al.[94]	1	f	58	2	n	l			AS				53	no		
Sipahioğlu et al. [95]	1	m	23	24	d				AS				36	no		
Snook [96]	2	f	82	12	d			yes	open			RT	18	yes		yes
		f	84	1	d			yes	conservative			RT	24	residual		yes
Sotje et al. [97]	1	m	5		n											
Stadelmeier et al.	4	f	54	31	n	l	yes		AS + open	yes			40	no		
		f	24	27	d	r			open				39	no		
		m	17	12	d	r			open			RSO	48	yes	open, AS, RSO	
		m	60	60	d	r		yes	open			RT	233	residual		yes
Tang et al. [98]	1	f	74		d	r							36	yes		
Tong et al. [99]	1	m	51	6	d		yes									
Toro et al. [50]	1	m	39	6	d	l		yes	open		aTSA		48	no		
Vj et al. [46]	6	f	42			l			AS				36	no		
		f	38			r			AS				36	no		
		m	74			r			AS				36	no		
		f	63			l			AS				36	no		
		f	60			r			AS				36	no		
		m	27			r			AS				36	no		
Zhao et al. [100]	1	f	7	6	d	b (mf)		yes	conservative							yes

(Abbreviations: m = male; f = female; n = nodular; d = diffuse; l = left; r = right; b = both shoulders; mf = multifocal; AS = arthroscopy; HSA = hemi shoulder arthroplasty; AP = arthroplasty (not specified); rTSA = reverse total shoulder arthroplasty; aTSA = anatomic total shoulder arthroplasty; RSO = radiosynoviorthesis; RT = radiotherapy)

.

## Discussion

### Main findings

This case series demonstrates that shoulder TGCT can be effectively managed with arthroscopic and open synovectomy combined with rotator cuff repair, achieving excellent long-term functional outcomes and quality of life. All patients maintained adequate pain control and returned to work or sports. Even the patient with most extensive intraosseous involvement showed disease stability over 10 years without revision surgery, highlighting the feasibility of conservative management after initial resection.

### Epidemiology

With a mean age of 38.8 ± 21.4 years (range 17–60 years) at their initial presentation, patients in this study fit well compared to other studies. It is suggested that the incidence peak is between 30 and 50 years of age [43]. Other literature reviews even suggest 50–80 years [44]. So far, only two case reports mentioned TGCT in the shoulders of adolescents [36, 45]. Females and males are equally affected [43].

### Diagnosis

In this case series, the mean duration from symptom onset to diagnosis was 32.5 ± 17.4 months, compared to 23.6 ± 28.4 months in the literature, highlighting a significant diagnostic delay in TGCT. This delay often results in advanced joint involvement, with approximately one-third of patients already exhibiting bone erosions due to synovial inflammation, mechanical stress from the lesion, or massive rotator cuff tears [41]. Despite this, the majority of the patients achieve symptom resolution after arthroscopic or open surgery [41, 43]. TGCT should be considered in young patients with unexplained shoulder pain and MRI findings suggestive of neoplastic disease [36, 37].

## Functional outcomes post-surgical intervention

There is limited literature on PROMs after TGCT surgery of the shoulder. VJ et al. reported on six patients undergoing arthroscopic synovectomy, showing significant improvements: Constant score from 64.8 to 94.5, American Shoulder and Elbow Surgeons (ASES) score from 81.2 to 99.7, and University of California, Los Angeles (UCLA) score from 23.2 to 34.8 at ≥ 36 months follow-up [46]. 

### Quality of life post-surgical intervention

According to our literature research this is the first study reporting quality of life results for patients with TGCT of the shoulder. Most patients reported outcomes equal to or exceeding the sex- and age-adjusted normative data for the German population [47]. Patient 1 developed severe rheumatoid arthritis, which likely accounts for the self-reported impairment in quality of life, despite demonstrating excellent functional outcomes for the shoulder. Patient 4 reported diminished physical functioning (Score = 50) compared to the age- and sex-adjusted German normative data (Mean Score = 76.4; 95%-CI = 73.7–79.1). This reduction may be attributable to his limited shoulder mobility. Patient 3 reported significantly worse general health (Score = 40) relative to the German normative population (Mean Score = 75.3; 95%-CI = 73.7–77.0). As the youngest participant in this study, the impact of impaired functionality may be more pronounced, particularly due to the inability to engage in sports and strength training, as reflected by a DASH sport/instrument score of 25. Furthermore, the most recent revision surgery occurred only 10 months prior to the assessment, suggesting potential for further improvement in personal strength and overall functional outcomes.

### Treatment – open surgery or arthroscopy?

There are no standardized guidelines for choosing between open and arthroscopic surgery in the management of shoulder TGCT. Both approaches yield good outcomes. Open synovectomy may be necessary for complete resection, especially in D-TGCT [42], while arthroscopic resection can also be effective if total synovectomy is achieved [39].

Arthroscopy offers the advantage of joint-wide visualization and lower morbidity and may be preferable when diagnosis is uncertain [27, 38]. In this series, one patient was successfully treated arthroscopically for re-recurrence. Alternating between open and arthroscopic techniques may help in recurrent cases.

Expert consensus by Stacchiotti et al. recommends macroscopic complete resection for all TGCT types, with arthroscopic or open approaches chosen, based on disease extent. D-TGCT has a high risk of local recurrence and should be discussed in a multidisciplinary tumor board. Extra-articular D-TGCT may require marginal resection through extended incisions due to soft tissue involvement [5]. 

#### What to do with rotator cuff tear?

In cases involving the shoulder, a thorough evaluation of the rotator cuff and articular surfaces is essential, as these factors play a critical role in determining clinical outcomes. Rotator cuff tears associated with shoulder TGCT are typically classified as massive, and degenerative changes in the articular surfaces are consistently observed to varying degrees. Consequently, in addition to synovectomy, the following surgical interventions are recommended if needed [40]: arthroscopic debridement and of the cuff tear [39, 41], partial cuff repair [41], HA [48, 49], and TSA [50].

Jong-Hun Ji et al. and Li et al. reported favorable clinical outcomes following arthroscopic or open synovectomy combined with rotator cuff repair, highlighting the therapeutic benefit of early intervention [27, 51]. Notably, Gumina et al. found that patients with D-TGCT who underwent arthroscopic synovectomy and subacromial debridement had significantly worse clinical outcomes compared to a control group without synovitis or osteoarthritis (arthroscopic debridement for irreparable rotator cuff tear alone). These poorer results were attributed to secondary osteoarthritis caused by unrecognized or late-treated TGCT [40]. Importantly, in cases where TGCT was identified early and treated with combined synovectomy and rotator cuff repair, outcomes were satisfactory, and no osteoarthritic changes were observed at follow-up.

#### Severe joint destruction and arthroplasty

TGCT of the shoulder can lead to severe joint destruction, often presenting with polycystic changes in the humeral head [52]. Elevated intra-articular pressure contributes to localized bone resorption and cyst formation, which may be further invaded by hypertrophic synovium via cortical breaches, the chondro-osseous junction, or vascular foramina [36, 48, 53]. As the villonodular tissue expands, progressive intraosseous infiltration may occur.

In advanced cases with substantial joint degeneration, various forms of shoulder arthroplasty—including HA, aTSA, and rTSA—have shown favorable outcomes [48, 50, 54]. Case reports demonstrate that these procedures effectively relieve pain, restore joint function, and reduce recurrence risk by enabling more complete dorsal synovectomy after osteotomy of the humeral head [5, 50]. The choice of prosthesis should be guided by the extent of osseous destruction and the presence of rotator cuff pathology. Specifically, rTSA may be advantageous in patients with concomitant rotator cuff tears, while aTSA or HA are appropriate in cases with preserved cuff integrity [42, 43]. Current evidence supports arthroplasty as a viable treatment option in patients with TGCT of the shoulder and advanced joint damage, particularly in elderly individuals with degenerative changes [36, 50].

## Adjuvant therapies

Percutaneous radiotherapy can offer some relief of the symptoms and it can be beneficial as adjuvant treatment after surgical resection for residual lesions [28, 29]. However, due to the young age of most patients, it is rarely a primary alternative. For inoperable cases, systemic therapy is preferable, while surgical management with complete macroscopic resection remains the treatment of choice for D-TGCT, and early marginal excision is recommended for the nodular form [5].

Given that patients with TGCT are typically young, and the disease is non-life-threatening, long-term complications such as malignant transformation, fibrosis, joint stiffness, and other sequelae are of particular concern. The use of radiotherapy in selected cases lacking viable alternative treatment options remains a subject of ongoing debate [5].

Radiosynoviorthesis has not demonstrated efficacy in the management of D-TGCT and should not be employed as a substitute for incomplete surgical resection. Prospective studies are warranted to better define its potential therapeutic role in the treatment of TGCT [5].

## Recurrence

Reported LR rates of TGCT after treatment vary widely between 9% and 55%, influenced by nodular or diffuse disease, joint location and follow-up duration [55–57]. Most of the recurrencies are seen in the first 2 years after surgery. Recurrence in the shoulder is rarely reported, likely due to the overall low incidence of TGCT at this site [50]. In this literature review, the LR rate for shoulder TGCT was 10.6% (*n* = 7) and the rate for residual disease was 4.5% (*n* = 3) at a mean follow-up of 37.6 ± 36.2 months (range 6–233 months).

D-TGCT has a higher recurrence risk than the localized form, though data specific to the shoulder are limited [57]. Key risk factors include incomplete surgical resection, particularly with extra-articular or intraosseous involvement, as well as delayed diagnosis and long symptom duration. These factors contribute to more extensive synovial disease and increase the likelihood of residual tumor and recurrence. Moreover, revision surgery in recurrent TGCT cases carries a substantially elevated risk of further recurrence [5]. Careful preoperative MRI evaluation is essential to detect occult or discontinuously growing lesions that may be missed during standard surgical approaches [58].

The main limitation of this study is the small sample size, which is inherent to the rarity of TGCT of the shoulder. Nevertheless, the extended follow-up duration and the inclusion of both functional and quality‑of‑life outcome measures enhance the clinical relevance of the presented findings. Despite these limitations, the present series contributes one of the longest follow-up periods and most comprehensive functional assessments reported to date for this rare entity, thereby expanding the current evidence base. These observations permit cautious but meaningful conclusions regarding the management of shoulder TGCT and underscore the importance of individualized treatment planning and vigilant follow‑up.

## Conclusion

TGCT of the shoulder is a rare condition that requires an individualized and multidisciplinary approach. Both arthroscopic and open synovectomy can achieve favorable outcomes when complete resection is accomplished. Given the risk of recurrence, structured long-term follow-up remains essential. To further refine treatment algorithms and identify prognostic factors specific to shoulder TGCT, larger multicenter or registry-based studies with standardized outcome reporting are needed.

## Data Availability

No datasets were generated or analysed during the current study.
